# Environmental factors associated with the prevalence of ESBL/AmpC-producing *Escherichia coli* in wild boar *(Sus scrofa)*

**DOI:** 10.3389/fvets.2022.980554

**Published:** 2022-10-13

**Authors:** Taras Günther, Stephanie Kramer-Schadt, Marcel Fuhrmann, Vitaly Belik

**Affiliations:** ^1^Department of Biological Safety, German Federal Institute for Risk Assessment, Berlin, Germany; ^2^System Modeling Group, Institute for Veterinary Epidemiology and Biostatistics, Freie Universität Berlin, Berlin, Germany; ^3^Department of Ecological Dynamics, Leibniz-Institute for Zoo and Wildlife Research, Berlin, Germany; ^4^Institute of Ecology, Technical University of Berlin, Berlin, Germany

**Keywords:** antimicrobial resistance, One Health, wildlife, spatial analysis, *E. coli*

## Abstract

Antimicrobial resistances (AMR) in bacteria, such as ESBL/AmpC-producing *E. coli*, are a burden to human and animal health. This burden is mainly driven by the consumption and release of antimicrobial substances into the environment. The pollution and contamination of habitats by AMR in bacteria and antimicrobial substances can lead to the transmission of bacterial AMR to wildlife. Therefore, it is necessary to understand the transmission cycle of antibiotics and resistant bacteria between humans, and animals as well as their occurrences in the environment. Environmental factors associated with the occurrence of bacterial AMR in wildlife can lead to a better understanding of the distribution of bacterial AMR in humans and animals using One Health approaches. Here, we analyzed data gathered in the framework of the German zoonoses monitoring program in 2016 and 2020 using spatiotemporal statistics to identify relevant environmental factors (e.g., livestock density, climatic variables, and human density) in association with the spatial distribution of ESBL/AmpC-producing *E. coli*. For this purpose, we developed a generic data integration and analysis pipeline to link spatially explicit environmental factors to the monitoring data. Finally, we built a binomial generalized linear mixed model (GLMM) to determine the factors associated with the spatial distribution of ESBL/AmpC-producing *E. coli*. In 2016 and 2020, 807 fecal samples from hunted wild boar (*Sus scrofa L*.) were randomly taken in 13 federal states and selectively analyzed for ESBL/AmpC-producing *E. coli*. Forty-eight isolates were identified in 12 German federal states, with an overall prevalence of 6%. We observed an almost three times higher probability of ESBL/AmpC-producing *E. coli* isolates in wild boar in counties with high cattle densities (OR = 2.57, *p* ≤ 0.01). Furthermore, we identified a seasonal effect in areas with high precipitation during the off-hunting seasons (OR = 2.78, *p* = 0.025) and low precipitation throughout the years (OR = 0.42, *p* = 0.025). However, due to the low amount of identified isolates, confidence intervals were wide, indicating a high level of uncertainty. This suggests that further studies on smaller scales need to be conducted with multiannual data and improved metadata, e.g., on the location, the hunting procedure, and species characteristics to be collected during field sampling.

## Introduction

Antibiotics are used for the treatment of most bacterial infectious diseases and are an important instrument in veterinary and human medicine. However, the consumption and release of antimicrobial substances into the environment can foster the development of antimicrobial resistance (AMR) in bacteria (1–4). While the development of resistances is an evolutionary process in bacteria known as the “arms race” to survive, the overconsumption and use of antimicrobial substances can further increase the selection pressure in bacteria to develop resistances against several antibiotics (2, 5, 6). This leads to a high exposure of bacterial AMR in humans, animals, and the environment (2, 3, 7, 8). In 2019, approximately 4.95 million people died as a direct consequence of infections with antimicrobial-resistant bacteria worldwide (8). Therefore, one key element to prevent the uncontrolled development of AMR in bacteria is to understand the transmission cycles of antibiotics and resistant bacteria between humans and animals, as well as their occurrences in the environment.

The role of antimicrobial-resistant bacteria in wildlife and the environment is of particular interest for One Health approaches considering the human-wildlife-livestock interface. Various studies show that multiple antimicrobial-resistant bacteria can be found globally in wildlife and the environment, i.e., soil, water bodies, feed, and food (1, 3, 9–13). In recent years, major efforts have been made to identify the reservoirs and sources of antimicrobial-resistant bacteria in wildlife.

In 2019, Torres et al. (12) highlighted that antimicrobial-resistant bacteria are not ubiquitously distributed among wildlife species. In particular, the wild boar (*Sus scrofa*) was suggested as an appropriate sentinel species to investigate the distribution and transmission cycles of antimicrobial-resistant bacteria in wildlife and the environment due to their omnivorous feeding habits and their high abundances in various habitats (12, 14, 15). The transmission of antimicrobial-resistant bacteria and antimicrobial residues to wild boar is most likely caused through the consumption of contaminated feed and water. Various studies have identified different sources as the origin of the occurrence of AMR in bacteria and antibiotics in the environment, such as manure-based fertilizers in agriculture applied to fields or pastures, sewage wastewater treatment plants, landfills, waste and aquacultural facilities (6, 16–18). Therefore, environments characterized by livestock farming and densely populated areas are often more predisposed to be contaminated by antimicrobial-resistant bacteria and antimicrobial substances, increasing the exposure of wildlife species living in proximity to these areas, such as wild boar (7, 11, 15, 19, 20).

However, the amount, composition, and durability of antimicrobial-resistant bacteria and antimicrobial substances in these environments often depend on additional environmental influences. In sewage treatment facilities, for example, the amount and composition of microorganisms, including antimicrobial-resistant bacteria, depends on different climatic factors, such as temperature (21). Furthermore, facilities such as hospitals, slaughterhouses, and residential areas in the catchment area of sewage plants can release high amounts of antimicrobial-resistant bacteria and antibiotics into sewage. This contaminated sewage can then enter nearby water bodies, such as rivers, due to insufficient filtration (4).

In livestock farming environments the accumulation of antimicrobial substances and antimicrobial-resistant bacteria is even more complex and influenced by climate, weather, soil properties and farm management, as well as the types of farm animals (22). For example, the storage conditions of manure and the timing of fertilization can have a significant effect on the amount of antimicrobial-resistant bacteria that reach fields and pastures (23). In the agroecosystem, environmental factors such as soil composition, local weather conditions, adjacent drainage ditches and habitats are also important factors leading to the accumulation of antimicrobial substances and antimicrobial-resistant bacteria in the environment (22). In addition, wild boar ecology needs to be considered; as social group living species they might serve as mobile links for AMR in bacteria (24) which is another possible driver of spread. This leads to a complex and dynamic transmission cycle, causing the presence of antimicrobial-resistant bacteria in wildlife species such as wild boar.

The German Federal Institute for Risk Assessment (BfR), the German Federal Office for Consumer Protection and Food Safety (BVL) and the German Federal States conduct routine monitoring programs for zoonoses in Germany. Due to the role of wild boar for game meat production, in 2016 and 2020, the German monitoring program included wild boar as a target species, testing for antimicrobial-resistant bacteria and other zoonotic agents (25–27). During the monitoring programs, 807 fecal samples of hunted wild boar (2016 *n* = 547 samples, 2020 *n* = 260 samples) were randomly taken across Germany and specifically tested for the presence of “extended spectrum (ESBL) and ampicillin class C (AmpC) beta-lactamase-producing *Escherichia coli*” (in the following: ESBL/AmpC *E. coli*) (25–27). The distribution pattern of ESBL/AmpC *E. coli* isolates seemed to be regionally concentrated and raised the question of whether there were spatiotemporal differences between isolates and negative samples.

In this study, we hypothesized that anthropogenic and environmental factors within the sampling regions could serve as indicators for the complex transmission cycle of ESBL/AmpC *E. coli* in wild boar. Regional density of livestock or human population might be an indicator for the exposure of the environment to contaminated sewage or fertilizers, and further, a possible transmission to wild boar. Here, we used the data of the German zoonoses monitoring program from 2016 and 2020 to develop an analysis pipeline that can be used in upcoming years as a tool for a standardized analysis (25, 27). The pipeline extracts and links environmental data to the sampling location and tested samples on ESBL/AmpC *E. coli*. For this purpose, a review of scientific literature was conducted to identify suitable environmental factors. Based on these results, the relevant environmental data were gathered from public online data portals.

Thus, the primary objectives of this study were i) to link data of the monitoring program with spatially related environmental factor data that were collected from different public online data sources, ii) to analyze the associations between the occurrence of ESBL/AmpC *E. coli* in wild boar and the environmental variables *via* geospatial analysis, and iii) to propose recommendations on data collection for monitoring of antimcirobial-resistant bacteria in wildlife using wild boar as a study model. This study will support a better understanding of the role of antimicrobial-resistant bacteria in wildlife and the environment following the framework of One Health.

## Materials and methods

### Sampling data and study area

**Figure 1:**
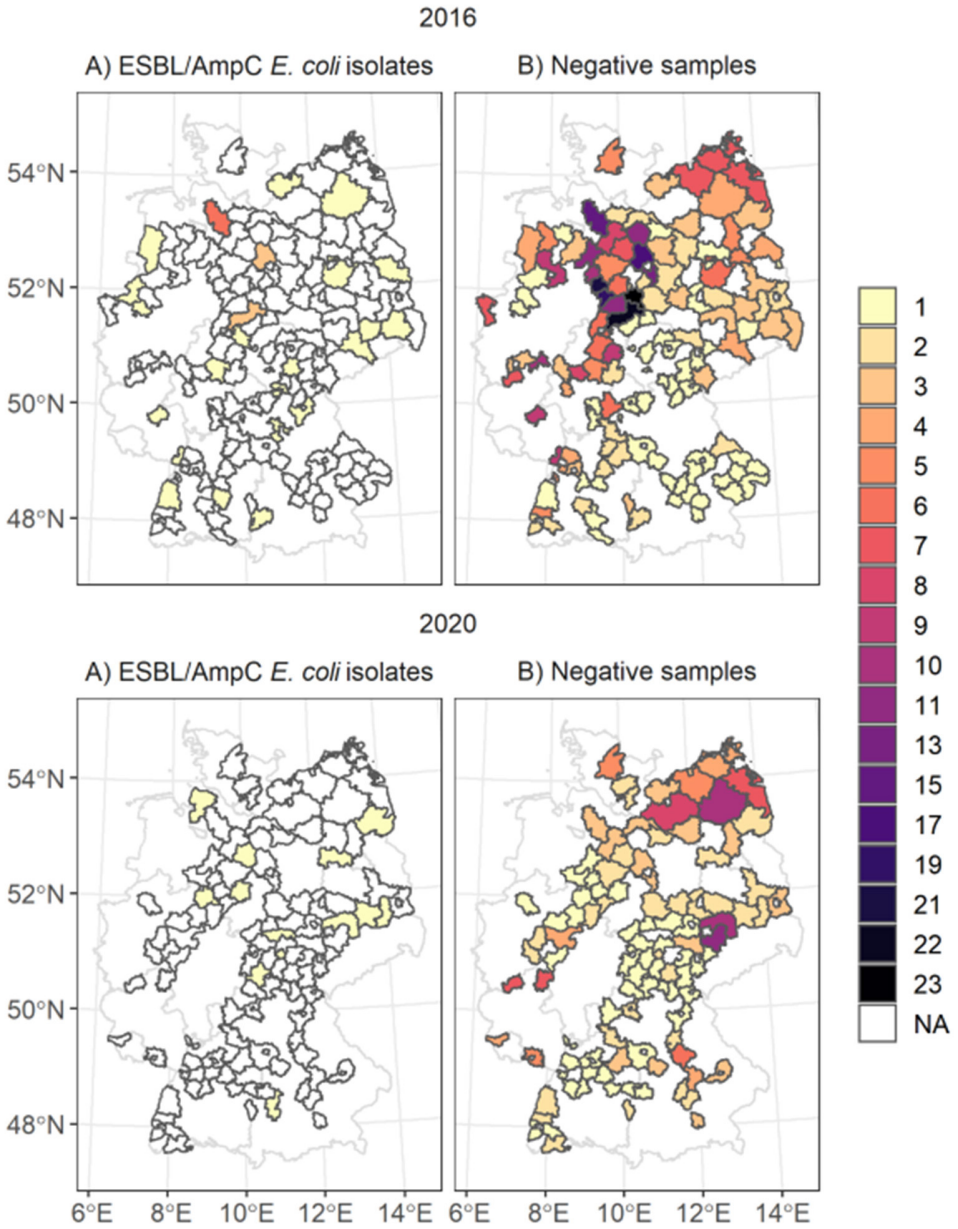
Spatial distribution of ESBL/AmpC *E. coli* isolates **(map A)** and negative samples taken **(map B)** in the counties. The color of the polygons represents the number of **(A)** number of isolates taken and in **(B)** the number of negative samples taken. The white areas marked as NA represent areas with no isolates identified.

In the German zoonoses monitoring programs in 2016 and 2020, 899 fecal and nasal swab samples from hunted wild boars were randomly collected and tested for different zoonoses and antimicrobial-resistant bacteria (25–27). In 2016, 547 out of 551 fecal samples of wild boar were tested specifically for ESBL/AmpC *E. coli*. In 2020 260 fecal samples out of 384 were tested specifically for ESBL/AmpC *E. coli*. The samples were collected in 14 out of the 16 German federal states, excluding Bremen, and Hamburg. We decided to exclude five samples taken in 2016 in Berlin since the provided information on the hunting area was not sufficient for conducting analyzes and because Berlin was the only sampling area comprising a metropolitan area. The minimum sample size defined for the monitoring programs was based on the hunting bags of the federal states 2013/2014 for the monitoring program 2016 and the hunting bags of 2017/2018 for the monitoring program 2020 (25, 27). However, the sample collection within the different federal states was not mandatory and depended on the availability of samples (Figure 1). The sample size differed from 1 to 23 per sampled county. The samples were taken monthly in 2016 and 2020, with a focus during the hunting seasons in the winter months of January, February, October, November, and December, where 78% of all samples were taken. Therefore, we defined two main seasons: the first season is defined as main hunting season (October–February) and the other one as the off-hunting season (March–September). The sampling locations were notified to the authorities at municipality or county level. Unfortunately, the samples were collected with the intention of aggregating the monitoring data at federal and national level. Therefore, detailed species information such as sex and age of the shot animals, as well as the exact locations, were often not concise or sufficiently reported. Furthermore, no information was provided on the hunting method within the different sampling regions. The results of the zoonoses monitoring programs have previously been published by Plaza-Rodriguez et al. (26) and in the report on the respective zoonoses monitoring in 2016 and 2020 (25, 27). These studies describe their findings on a national level for different animal species and zoonotic bacteria. Our study is based on the raw data of the monitoring program focusing on the wild boar samples tested for ESBL/AmpC *E. coli*. We extended the reported data with additional information to allow detailed spatial analysis.

### Laboratory analysis

The primary isolation of ESBL/AmpC *E. coli* in collected fecal samples was carried out in accredited state laboratories according to the EU reference laboratory protocol for the isolation of ESBL-, AmpC- and carbapenemase-producing *E. coli* in caecal samples (28). These results were reported to the German Federal Office for Consumer Protection and Food Safety (BVL) for aggregation and reporting at national level. The confirmation, characterization, and phenotypic resistance testing of ESBL/AmpC *E. coli* isolates was performed at the National Reference Laboratories for Antimicrobial Resistance (NRL-AR) at the BfR. The antimicrobial susceptibility testing (AST) was conducted with broth microdilution method according to CLSI M07-A10 and CLSI M45-A, using the standardized EUVSEC and EUVSEC2 plates (TREK Diagnostic Systems) for 14 antibiotics (Commission Implementing Decision (CID) 2013/652/EU) (26). A further characterization in regard to harbored ESBL/AmpC genes within *E. coli* isolates resistant to third generation cephalosporins was conducted in three steps (26). First, a prescreening by real-time PCR was performed for the detection of the typical beta-lactamases TEM, CTX, SHV, and CRY (26). Thereafter, the PCR products were analyzed by Sanger sequencing for the determination of the ESBL variant (26). In a third step, isolates that were negative with real time PCR were screened by PCR for the presence of *bla*_*FOX*_*, bla*_*MOX*_*, bla*_*CIT*_*, bla*_*DHA*_, and *bla*_*EBC*_ genes (26). Since some discovered beta-lactamase types differed within the primer regions, it was not possible to distinguish between CTX-M-14 and -*17* (CTX-M-14 like), between CTX-M-65 and 90 (CTX-M-65-like), and between CMY-2/-22 and−66 (CMY-2-like) (26). The detailed laboratory analysis is already published in Plaza-Rodriguez et al. (26). We used the discovered resistance genes in our study to look for specific spatial patterns for further validation of the origin of resistances.

### Data analysis pipeline

**Figure 2:**
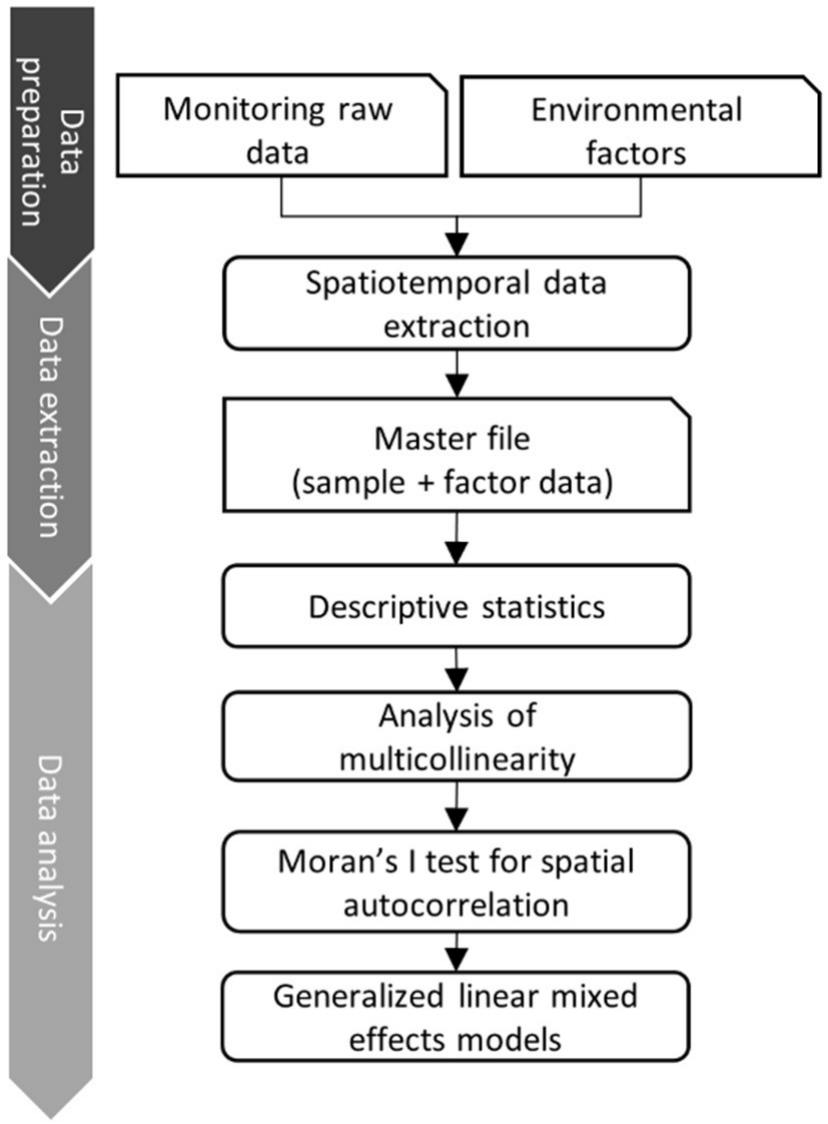
Data analysis pipeline that combines monitoring raw data with data of explicit environmental factors (right vertical axis). On the left the 3 main steps of the data pipeline are described.

The data analysis pipeline (Figure 2) consists of three main steps: i) data cleaning, ii) data linking and extraction of environmental predictor variables and iii) statistical data analysis (29). During the data cleaning step, we deleted duplicated data from the same wild boar sample. Subsequently, the information on the sample origin was merged according to the county code with the spatial vector data of the Federal Agency for Cartography and Geodesy (BKG, scale 1: 250,000) (30). This vector data contains the polygon information of the borders of all counties of Germany. Data on explicit environmental factors were collected from different public data sources and formats (raster, csv and Excel). Tabular data were linked based on the reported county keys. We harmonized raster datasets using Lambert azimuthal equal-area projection (LAEA: EPSG-Code 3035). The template raster resolution was 100 square meters (m^2^), which represents the specific information about the transformed data set in each grid cell. The processed environmental raster files were stacked and extracted according to the reported counties, months, and years using the “*exactextractr*” R-package (31). The data analysis pipeline was developed in R 4.1.1 (32), combining all steps in one code base. We used, among others, the packages *raster, sf* , *exactextractr* and *tidyverse* (31, 33–35).

### Environmental predictor variables

We based the selection of environmental predictors on a review of current scientific literature focusing on the development and contamination of bacterial AMR in the environment. In several publications, the role of agriculture and densely populated areas are described, but also climatic factors such as high temperatures and precipitation (11, 12, 15, 20–22, 26). Therefore, we collected meteorological, agricultural and geographical data from publicly available websites listed in Table 1. The meteorological data originate from the “Deutscher Wetterdienst” (DWD), a federal authority that is responsible for weather and climate information in Germany (36). We derived monthly information on air temperature (measured in 2016 and 2020 in 2 m height) and precipitation (from 2016 and 2020). We also included the climatic precipitation and temperature (both measured and averaged from 1991 to 2020) to identify weather anomalies during the years 2016 and 2020. This information was provided as raster files for each month and year. We projected the raster files into the target LAEA projection (see Section 2.3), and the values of the weather data were extracted as median values for each notified county of origin and the month of sampling. Information on the human population density and livestock production systems in 2016 and 2020 were collected from the data portal “www.regionalstatistik.de” provided by the German federal statistical offices. We downloaded information on the human population density, cattle density, and pig density per km^2^ in each county from the “Regional Atlas of Germany” as csv files. The downloaded data were merged according to the county code (NUTS) with the monitoring data. To account for the transmission of ESBL/AmpC *E. coli* within the wild boar population, we used raster data of the MaxEnt model prediction based on presence-background data describing suitability for wild boar occurrence (2014–2017) published by the *ENETWILD*-consortium 2019 (37). The wild boar presence probability raster data was also reprojected into the target LAEA projection and extracted as median for each county (see Section 2.3). Thus, all data sources were summarized in one master file that contained the information on ESBL/AmpC *E. coli* isolates obtained from fecal samples, the environmental variables and the spatial information given as the geometry of the county.

**Table 1:** Collected data and their origin.

**Category**	**Factors**	**Unit**	**Year**	**Source**	**Reference**
Weather/Climate	Average monthly temperature	Degree Celsius	2016, 2020	*DWD*	cdc.dwd.de
	Average monthly precipitation	mm	2016, 2020	*DWD*	cdc.dwd.de
	Average monthly temperature (climate) (1991–2020)	Degree Celsius	1991–2020	*DWD*	cdc.dwd.de
	Average monthly precipitation (climate) (1991–2020)	mm	1991–2020	*DWD*	cdc.dwd.de
Sources/Proxys	Human population density	Human population density/ Km^2^	2016, 2022	*Regionalstatitik.de*	www.regionalstatistik.de
	Cattle density	Cattle density /Km^2^	2016, 2020	*Regionalstatitik.de*	www.regionalstatistik.de
	Pig density	Pig density /Km^2^		*Regionalstatitik.de*	www.regionalstatistik.de
Wild boar	Wild boar presence probability	Suitability (Low:0/High:1)	2014–2017	*ENETWILD*-consortium	https://enetwild.com/maps/
Geographic	County Germany GE250 data base		2016	*BKG*	www.bkg.bund.de/
Time	Month		2016	Raw Data	
	Main hunting season and off-hunting season			generated	

### Statistical analysis

The statistical analysis was performed in four main steps. We first conducted a descriptive analysis of the spatial distribution of samples. Before model fitting, we tested all extracted environmental variables for multicollinearity with the Spearman's rank correlation test (“*ggally”* R-package) and kept all variables with |*rho*| < 0.7 (Supplementary Figure 1). We tested the samples taken on spatial autocorrelation using Moran's I, which relies on a comparison of the distance between the center points of each county. All counties sampled were spatially independent (*p* = 0.01). Next, we used generalized linear mixed-effects models (GLMM) with the presence or absence of ESBL/AmpC *E. coli* represented as binary response variable (1/0), with logic link function, binomial error distribution. As explanatory predictor variables, we included the environmental factors (covariates) such as cattle density, pig density, human population density, the average monthly precipitation, the climatic mean precipitation, the wild boar presence probability, and the average monthly temperature. All explanatory predictor variables were normalized using the scale function to account for different units. To assess the temporal dynamics of the occurrences of ESBL/AmpC *E. coli* in wild boar, we included season as an interaction term combined with each environmental covariate. To account for regional and yearly differences, we used the county ID as well as the year as random effects. We ran the GLMM using the R-package “*glmmTMB*” (38). Estimates were transformed to odds ratios (OR) with the *sjPlot* package (39). The standard significance threshold was set to *p* < 0.05 (95% confidence interval, CI). Finally, we examined the model for linearity of predictors, independence of errors and dispersion with the *DHARMa* package (40) (Supplementary Figures 2, 3).

## Results

### Spatial distribution of the samples

We linked and analyzed the data of 802 fecal samples of dead wild boars tested for ESBL/AmpC *E. coli* as part of the zoonoses monitoring program in 2016 and 2020. The sample size differed between the years: for 2016, 542 samples, and for 2020, 260 samples. The samples originated from 181 German counties in 13 federal states (Figure 1). Most samples, were collected in the federal state of Lower Saxony in the northwestern part of Germany (*n* = 271, 33.6%). At the county level, most fecal samples were collected in 2016 in Goslar, where all 23 samples were negative. In 2020, most samples originated from the federal state Mecklenburg-Western Pomerania (*n* = 40) and the counties Leipzig (*n* = 11) and North Saxony (*n* = 11) (Saxony). Most samples tested for ESBL/AmpC *E. coli* (78.4%) were taken during the main hunting season, especially in November (56%). During the off-hunting season (March-September), 15.8% of the samples were taken.

A total of 48 ESBL/AmpC *E. coli* isolates out of 802 fecal samples were detected in 39 counties and 11 federal states (Figure 1). In 2016, 37 isolates out of 544 samples and in 2020, 13 isolates out of 260 samples were identified. The highest number of ESBL/AmpC *E. coli* isolates in 2016 were found in the federal state Lower Saxony with 13 isolates out of 241 fecal samples and on the county level in Rotenburg (Wümme) (5 isolates out of 15 fecal samples) (Supplementary Figure 5). The distribution of the sample size and the sample regions differed between 2020 and 2016, and most isolates of 2020 were identified in the federal state Brandenburg, with four isolates. No county had more than one identified isolate in 2020.

**Figure 3:**
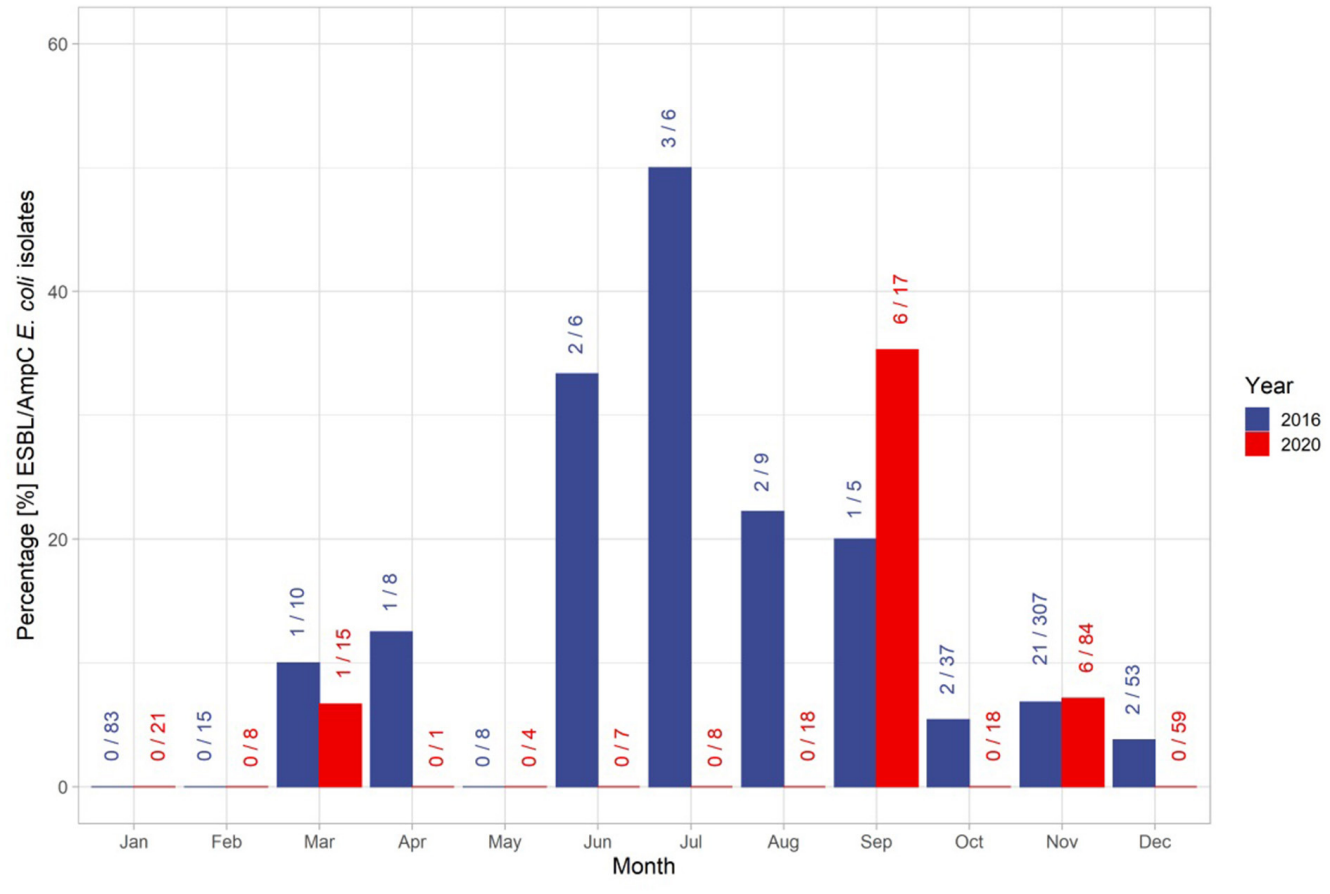
Distribution of isolates and the total number of samples throughout the years 2016 and 2020. The first number at each bar represent the number of identified ESBL/AmpC *E. coli* isolates within the month, and the second number after the slash sign represents the total number of samples taken within the month. The colors of the bars and numbers represent the associated year.

The overall temporal distribution of the taken samples was characterized by the hunting season (Figure 3). In November 2016, 22 isolates were found in the 306 samples taken, which is the highest number of isolates found. In January (*n* = 83), February (*n* = 15) and May (*n* = 8) no isolates were identified. The overall proportion of identified isolates during the off-hunting season was 30% higher than in the main hunting season. However, the samples taken during the off-hunting season represented only 16% of all collected samples (Figure 3). The proportion of isolates ranged from 33% (*n* = 6, isolates = 2) in June, 50% in July (*n* = 6, isolates = 3), 22% in August (*n* = 9, isolates = 2) and 25% in September (*n* = 4, isolates = 1) (Figure 3). In 2020, isolates were identified only in 3 months: March, September, and November. In September (*n* = 6) and November (*n* = 6) 12 out of 13 identified isolates were found. Hence, 53% of all isolates were identified within the off-hunting season.

In 2016, 25 isolates of the 35 identified ESBL/AmpC *E. coli* isolates were additionally tested for their resistance genes. Fifty-seven percent of the isolates were contained *bla*_CTX − M−1_ (*n* = 11) genes, 17% *bla*_CTX − M−15_ (*n* = 5) genes and 13% as *bla*_CTX − M−14_ -like genes (*n* = 3). Furthermore, one isolate contained *bla*_CTX − M65_, and another isolate was classified as AmpC phenotype *bla*_CMY2_-like gene. Sixty-four percent of the 25 analyzed isolates were identified during the hunting season and 28% during the off-hunting season. Most isolates identified in 2016 contained *bla*_CTX − M−1_ genes, with nine isolates in the main hunting season and four in the off-hunting season. At the county level, most ESBL/AmpC *E. coli* were identified during the main hunting season in Rotenburg (Wümme) in Lower Saxony, with the ESBL genes *bla*_CTX − M−1_ (*n* = 3) and *bla*_CTX − M−15_ (*n* = 2). Most of the analyzed resistance genes (40%) originated from the federal state of Lower Saxony. The resistance genes *bla*_CTX − M−1_ (*n* = 5), *bla*_CTX − M15_ (*n* = 3), *bla*_CTX − M−14_ -like (*n* = 1) and one *bla*_CTXM − M−65_ were identified in the isolates from Lower Saxony. These isolates were collected during the main hunting season. Most samples during the off-hunting season originated from the federal state of North Rhine-Westphalia, with one isolate detected with *bla*_CTX − M−1_ and another with *bla*_CTX − M−15_ collected during June 2016. Most samples in 2020 contained genes classified as *bla*_CTX − M−1_ (*n* = 6) and *bla*_CTX − M14_-like. In the other isolates, the AMR genes were classified as *bla*_CTX − M−15_, *bla*_CTX − M−27_, *bla*_SHV − 12_, and *bla*_TEM − 52 − B_.

### Environmental factors associated with the distribution of ESBL/AmpC *E. coli*

**Figure 4:**
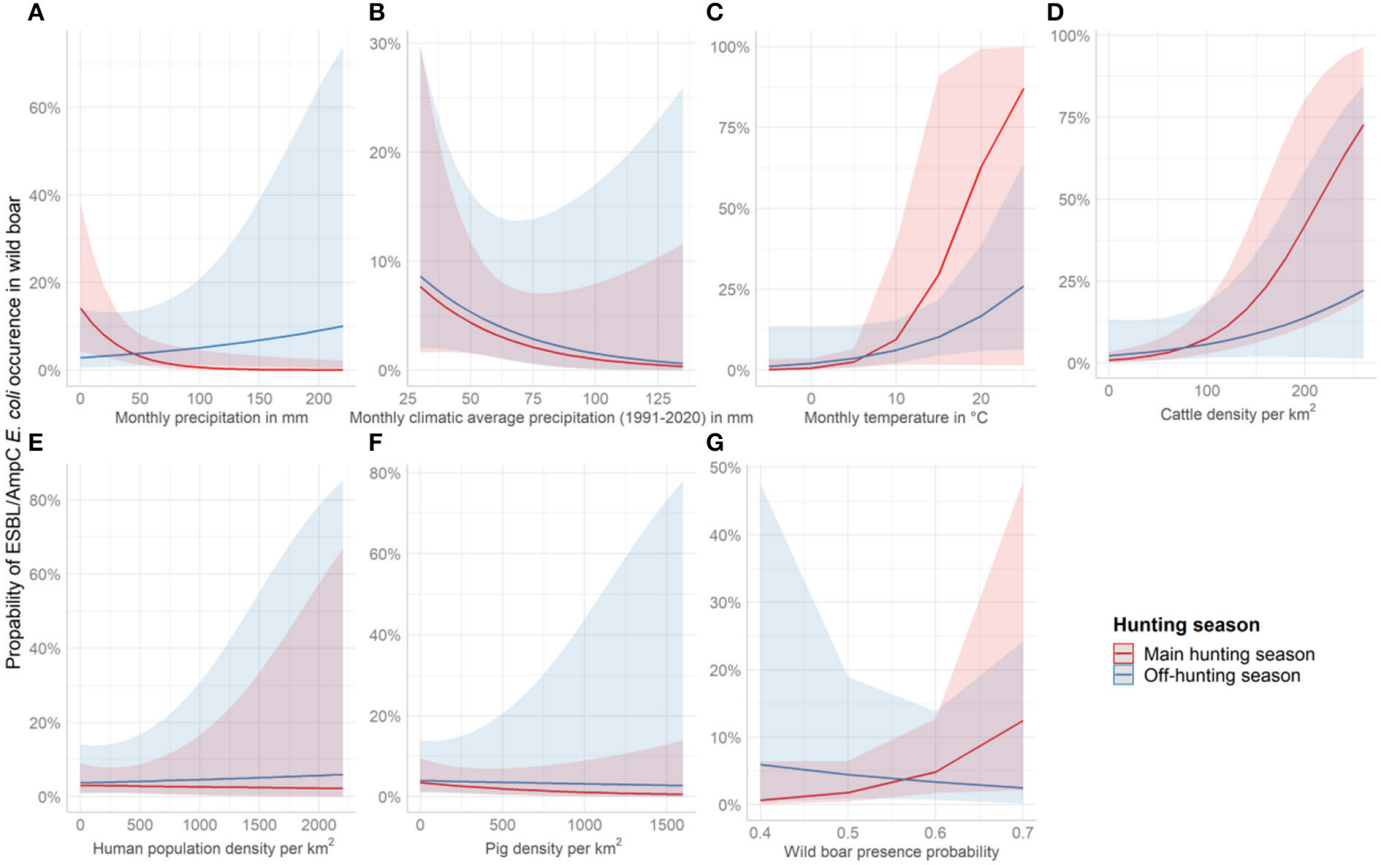
Effect plots showing the increase or decrease in the probability to identify ESBL/AmpC *E. coli* in wild boar per covariate and season. The red line represents the course of the main hunting season and the blue line the course of the off-hunting season. The covariates shown are **(A)** average monthly precipitation 2016 and 2020 in mm, **(B)** average monthly climatic precipitation (1991-2020) in mm, **(C)** average monthly temperature of 2016 and 2020 in °C, **(D)** cattle density per km^2^, **(E)** Human population density per km^2^, **(F)** pig density per km^2^, and **(G)** wild boar abundance.

ESBL/AmpC *E. coli* in wild boar was almost three times more likely (OR = 2.78, *p* = 0.03) in counties with high average precipitation during the off-hunting season. In contrast, the overall probability to identify ESBL/AmpC *E. coli* in areas with low monthly average precipitation was significant as a single effect (OR = 0.42, *p* = 0.02) (Figure 4A and Table 2). The climatic average precipitation showed no significant effect in our model (Figure 4B and Table 2). Furthermore, areas with a higher average monthly temperature showed an almost four times higher occurrence of ESBL/AmpC *E. coli* in wild boar (OR = 3.61, *p* = 0.06) (Figure 4C and Table 2). Nevertheless, this effect was not significant.

**Table 2:** Estimates of fixed effects influencing the presence of ESBL/AmpC *E. coli* in wild boar (*Sus scrofa L*.) with the county as random effect (significance threshold *p* < 0.05).

**Predictors**	**Odds ratios**	**CI**	***p***
(Intercept)	0.03	0.01–0.09	**<0.001**
Hunting season [Off-hunting season]	1.30	0.33–5.19	0.709
Average monthly precipitation in mm	0.42	0.21–0.87	**0.020**
Average monthly precipitation in mm (climate)	0.61	0.29–1.28	0.193
Human population density per km^2^	0.97	0.65–1.47	0.893
Average monthly temperature	3.61	0.92–14.18	0.066
Wild boar presence probability	1.55	0.91–2.62	0.104
Cattle density per km^2^	2.57	1.53–4.32	**<0.001**
Pig density per km^2^	0.79	0.51–1.23	0.293
Hunting season [Off-hunting season] * Average monthly precipitation in mm	2.78	1.13–6.80	**0.025**
Hunting season [Off-hunting season] * Average monthly precipitation in mm (climate)	1.07	0.38–3.04	0.899
Hunting season [Off-hunting season] * Human population density per km^2^	1.07	0.61–1.89	0.810
Hunting season [Off-hunting season] * Average monthly temperature	0.46	0.11–1.97	0.299
Hunting season [Off-hunting season] * Wild boar presence probability	0.57	0.25–1.28	0.171
Hunting season [Off-hunting season] * Cattle density	0.59	0.25–1.39	0.226
Hunting season [Off-hunting season] * Pig density	1.21	0.58–2.54	0.618
**Random effects**	
σ^2^	3.29
τ_00__County_	0.20
τ_00__Year_	0.21
ICC	0.11
N _County_	180
N _Year_	2
Observations	802
Marginal R^2^/Conditional R^2^	0.357/0.429

The fixed effects explained ~ 35% of the variance (marginal R2), while adding the random structure explained another 7%.

In counties with a high cattle density, an almost three times higher (OR = 2.57, *p* = < 0.01) (Figure 4D and Table 2) probability of ESBL/AmpC *E. coli* occurrence in wild boar was estimated. However, no effect in interaction with the hunting season was observed (Figure 4D and Table 2). The human population density (OR = 0.97, *p* = 0.89) (Figure 4E and Table 2) and pig density (OR = 0.79, *p* = 0.29) (Figure 4F and Table 2) showed no significant association with the likelihood of ESBL/AmpC *E. coli* occurrence as single effects, as well as in interaction with the hunting seasons (Table 2). The wild boar presence probability estimated by the MaxEnt prediction of the *ENETWILD*-consortium showed a slight positive effect (OR = 1.55, *p* = 0.10) even though it was non-significant (Figure 4G and Table 2). The overall model explained 35% of the variance in the data, whereas another 7% was explained by the random effects, year and county.

## Discussion

The occurrence of ESBL/AmpC *E. coli* in wild boar feces had seasonal and spatial differences in 2016 and 2020 in Germany. We identified anthropogenic and seasonal effects such as cattle density, and precipitation associated with the occurrence of ESBL/AmpC *E. coli* isolates in wild boar.

In June 2016, the precipitation in the counties Borken and Recklinghausen was 120 mm higher than the climatic monthly average (1991–2020) (DWD) (Supplementary Figure 4). During that time, heavy rainfalls and floods were reported in the regions where the isolates were identified (41). These events and hence the significant model outcome indicated a positive association between high precipitation in summer 2016 and the occurrence of ESBL/AmpC *E. coli* in wild boar. The transmission might be caused by the overflow of sewage water in sewage water plants. This is one of the main sources leading to the uncontrolled spread of bacterial AMR and other zoonotic pathogens into water bodies (4). We assume that wild boars came into contact with contaminated sewage water or with water from rivers next to sewage plants where an overflow occurred. The genes *bla*_CTX − M−1_ and *bla*_CTX − M−15_ were detected in isolates taken during the flooding event in June 2016 in Borken and Recklinghausen in the federal state of North Rhine-Westphalia. *Bla*_CTX − M−1_ genes were previously found in non-clinical isolates of humans as well as in livestock (42), while *bla*_CTX − M−15_ genes are predominantly found in clinical isolates from humans (43). The region of Borken and Recklinghausen has a high number of human settlements, which supports the assumption of transmission from sewage to wild boar. This positive association is in line with other studies that demonstrated a connection between high human population density and bacterial AMR emergence (11, 12, 15, 26). However, in our model, the human population density did not show a positive association with the isolates identified, and we were unable to distinguish the effects of the human population density and the pig density on the ESBL/AmpC *E. coli* occurrences in wild boars within our model. In contrast, lower average monthly precipitations had a significant effect on the probability of ESBL/AmpC *E. coli* occurrence in wild boar for the whole year. This may indicate that single weather events can significantly change the outcome of the ESBL/AmpC *E. coli* occurrence in wild boar. For example, for 2020 we were unable to identify a difference in the average monthly precipitation and the average climatic monthly precipitation within the counties. Moreover, we saw an almost four times higher probability of ESBL/AmpC *E. coli* occurrence in wild boars hunted in counties with a higher average temperature. Even though this effect was not significant with a *p*-value of 0.07. Higher temperatures can enhance biological activity in soils and water and therefore might also influence the occurrence of AMR in bacteria in the environment (4, 18, 22). This can also lead to seasonal differences in the occurrence of ESBL/AmpC *E. coli* in sewage water of sewage cleaning plants (6). Even during winter, the occurrence in counties with milder temperatures was higher than in colder ones. However, in 2020 in May, June, and July no isolates were identified in the sampled areas with overall similar sample sizes compared with 2016.

Interestingly, the cattle density showed a highly significant association with the occurrence of ESBL/AmpC *E. coli* in wild boar. This is in line with the report of the zoonoses monitoring in 2015 and 2017, where cattle populations had a prevalence of 60 and 68% ESBL/AmpC *E. coli* isolates, respectively (44, 45). The high prevalence of ESBL/AmpC *E. coli* isolates in cattle populations also appears to impact the incidence of ESBL/AmpC *E. coli* isolates in wild boar, suggesting exposure from manure, farms, and cattle. In addition, 66% of the identified isolates were found during November. During this month, the spread of slurry is not allowed. However, prior to November, farmers attempt to apply manure to empty storage capacities before winter. This may lead to a temporary increased exposure of wildlife to manure contaminated with antimicrobial-resistant bacteria. Pastures were the only areas where fertilization was allowed until November 1st. Therefore, this would also mean that the prevalence of antimicrobial-resistant bacteria in wildlife would increase at the beginning of the vegetation period when farmers fertilize crops like wheat (*Triticum aestivum*) and barley (*Hordeum vulgare*) with nitrogen and slurry. In March, we saw in both years one ESBL/AmpC *E. coli* isolate in wild boars, but not during April and May. Another possible transmission route might be the release of contaminated sewage water from sewage plants, for example, from the slaughtering production industry releasing antimicrobial-resistant bacteria to the environment. Even though, this is not specific to the month of November itself (46, 47). To determine valid statements on the origin of the isolates, further studies are needed on smaller sampling areas to investigate such relations and to link, for example, genetic information of isolates identified in samples of sewage and farms with isolates of wild boar. Farm management might be another factor influencing AMR in bacteria release into the environment. Unfortunately, the monitoring data for 2016 and 2020 is too limited to provide information on the direct transmission of ESBL/AmpC *E. coli* between wild boar and cross-species transmission with other wildlife and livestock species. However, we observed a non-significant positive trend of ESBL/AmpC *E. coli* occurrence in areas with a higher wild boar presence probability by using the MaxEnt model prediction of the *ENETWILD*-consortium. This result may indicate that transmission of ESBL/AmpC *E. coli* may also be driven in part by the wild boar population itself, even though the association was non-significant. Accordingly, wild boar ecology needs to be considered in future studies to gain a better understanding of the transmission cycles of ESBL/AmpC *E. coli* within the wild boar population.

There are further limitations in this study to be acknowledged. We observed wide confidence intervals for all factors that indicate statistical uncertainties. Furthermore, there are possible biases in the sampling related to the non-reported hunting procedures. This, together with the low numbers of ESBL/AmpC *E. coli* isolates, might lead to confounding effects. Firstly, the hunting strategy itself might contribute to the unequal distribution of the samples throughout the years. While traditionally large drive hunts dominate in winter and whole sounders are shot, hide hunts in summer target single individuals (48). Repeated samples from the same sounder might lead to clustering, e.g., if the entire sounder had not encountered sources of bacterial AMR, the likelihood of finding many isolates might be on average lower and vice versa. Moreover, we need to consider that the samples in summer were only taken in few municipalities. For this reason, we can only speculate if the higher prevalence observed during the off-hunting season in 2016 (Figure 3) is truly representative or just subject to sampling bias, especially when considering the data of 2020, where only during May, September, and November isolates were identified, and the model outcome was not significant for the hunting season. Additionally, we cannot provide detailed information on how the sampling process was affected by the corona pandemic throughout 2020, which caused an overall smaller sample size during the year.

Not only was the sampling different throughout the years, but also in space. For example, the authorities in Lower Saxony took the required minimum sample of wild boar samples in 2016 in periods of time instead of during the whole main hunting season. Lower Saxony is one of the most important regions in Germany for livestock production. However, during the off-hunting season, the federal state of Lower Saxony was underrepresented in the samples taken, while during the winter of 2016 it was overrepresented (Supplementary Figure 5). Likewise, samples taken in areas with a high cattle density are temporarily underrepresented. This may bias our model results even though we integrated the sample region as a random effect. Therefore, further studies and upcoming monitoring programs of wildlife need a standardized annual data collection and should report information on the type of hunting as well as the exact locations.

Moreover, the livestock industry in our model is not completely represented. The poultry industry, one of the largest users of antibiotics in livestock farming, was not included in the model (49, 50). In the study of Urra et al. (23), the authors show that in fresh manure from chickens, high proportions of AMR in bacteria can be identified. It is then likely that poultry can contribute to the distribution of AMR in bacteria to wildlife as well. We were unable to identify suitable data sources on the density of poultry for the years 2016 and 2020 and therefore did not include this as a covariate. Thus, the poultry industry should also be considered in future studies to determine the impact of different livestock species on the distribution of antimicrobial-resistant bacteria in wildlife. A comparison of the spatiotemporal prevalence data of livestock with the data of wildlife species could be beneficial as well, to better understand the transmission pathways from agriculture to wildlife.

Overall, our data pipeline allowed an extension and spatiotemporal analysis of the monitoring data. The analysis reveals the value of linking different environmental open data sources with data of monitoring programs in order to determine effects that might be associated with the distribution of bacterial AMR in wildlife. Our study demonstrates that monitoring programs targeting the distribution of antimicrobial-resistant bacteria in wildlife require further adjustments regarding the metadata collected during sampling to account for biases. Food safety monitoring programs are often designed based on livestock and food products for aggregation on a national level, considering privacy protection. Therefore, metadata on the individual level such as information on the correct sampling location is limited. In the case of free-ranging wildlife, sufficient harmonized information on age, sex, hunting methods and the correct sampling location is important. Furthermore, regarding wildlife the report of national units such as municipality or county level is not as beneficial as the report of specific coordinates (longitudes and latitudes), where the animals were sampled. Accurate coordinates are much more flexible to use regarding data extraction, linking, and processing on different spatial scales. It would also improve the comparability with other studies in the field and retrospective analyses, even though analytical methods might differ (15).

## Conclusion

The distribution of ESBL/AmpC *E. coli* in wild boar is associated with environmental and anthropogenic factors. We showed that seasonal effects such as temperature and precipitation can have a significant association with the occurrence of ESBL/AmpC *E. coli* isolates in fecal samples from wild boar. Our results hint that weather events such as heavy rainfalls, floods, and high temperatures may increase the abundance of bacterial AMR in the environment, which is relevant to the potential effects of climate change. Interestingly, we also identified a positive effect on the distribution of ESBL/AmpC *E. coli* in wild boar by cattle density. This suggests that wild boars are exposed to antimicrobial-resistant bacteria in areas with large cattle populations. However, our analysis showed a high level of uncertainty, suggesting that multiannual data and small-scale studies in wildlife are needed to verify our findings.

## Data availability statement

The datasets presented in this article are not readily available because the data analyzed in this study is subject to the following licenses/restrictions: The dataset used in this article belongs to the German National Zoonoses Monitoring Program. Currently these data are not publicly available, however work is currently underway to create a public database that contains this data along with many other data from other programs and years. The developed data integration and analysis pipeline in R is not publicly available and still in development. Requests to access the datasets should be directed to taras.guenther@bfr.bund.de.

## Author contributions

TG, SK-S, MF, and VB: conceptualization and review and editing. TG and SK-S: methodology. TG: formal analysis and original draft preparation. All authors contributed to the article writing and approved the submitted version.

## Funding

This work was funded *via* the ORION project and the German Federal Institute for Risk Assessment (BfR). The ORION project is part of the European Joint Programme One Health EJP, which has received funding from the European Union's Horizon 2020 research and innovation programme under Grant Agreement No. 773830.

## Conflict of interest

The authors declare that the research was conducted in the absence of any commercial or financial relationships that could be construed as a potential conflict of interest.

## Publisher's note

All claims expressed in this article are solely those of the authors and do not necessarily represent those of their affiliated organizations, or those of the publisher, the editors and the reviewers. Any product that may be evaluated in this article, or claim that may be made by its manufacturer, is not guaranteed or endorsed by the publisher.
