# Supplementary material for: Environmental factors associated with the prevalence of ESBL/AmpC-producing Escherichia coli in wild boar (Sus scrofa)
Source: Front Vet Sci. 2022 Oct 13;9:980554. doi: 10.3389/fvets.2022.980554 (PMC9608181; doi:10.3389/fvets.2022.980554)
Supplement: Supplementary file 1 [file Data_Sheet_1.docx]

Supplementary Material

# Supplementary Figures


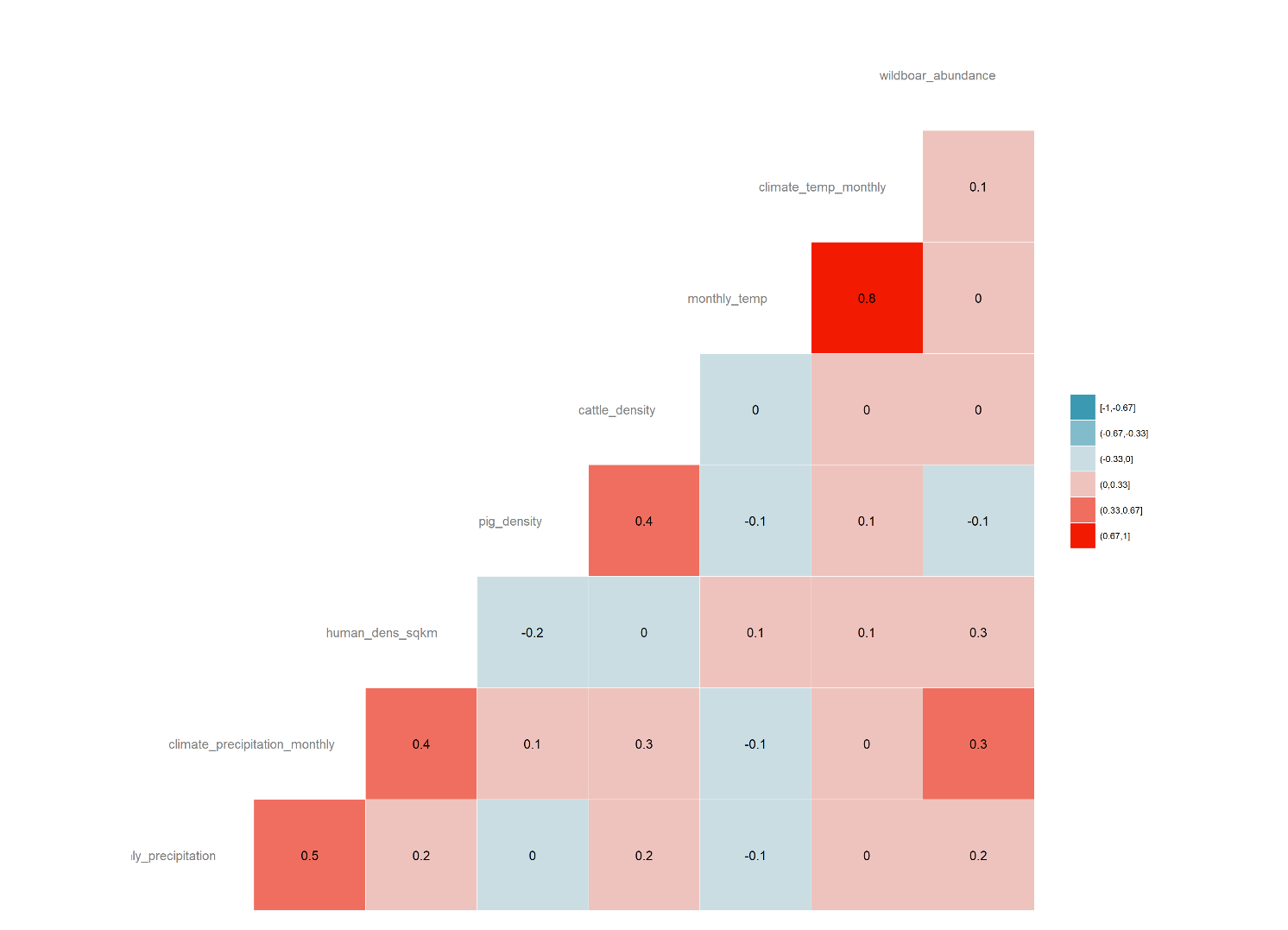


Supplementary Figure 1 Spearman’s rank correlation matrix showing the results of the test on multicollinearity for the selected environmental factors, which were used in the final equation.


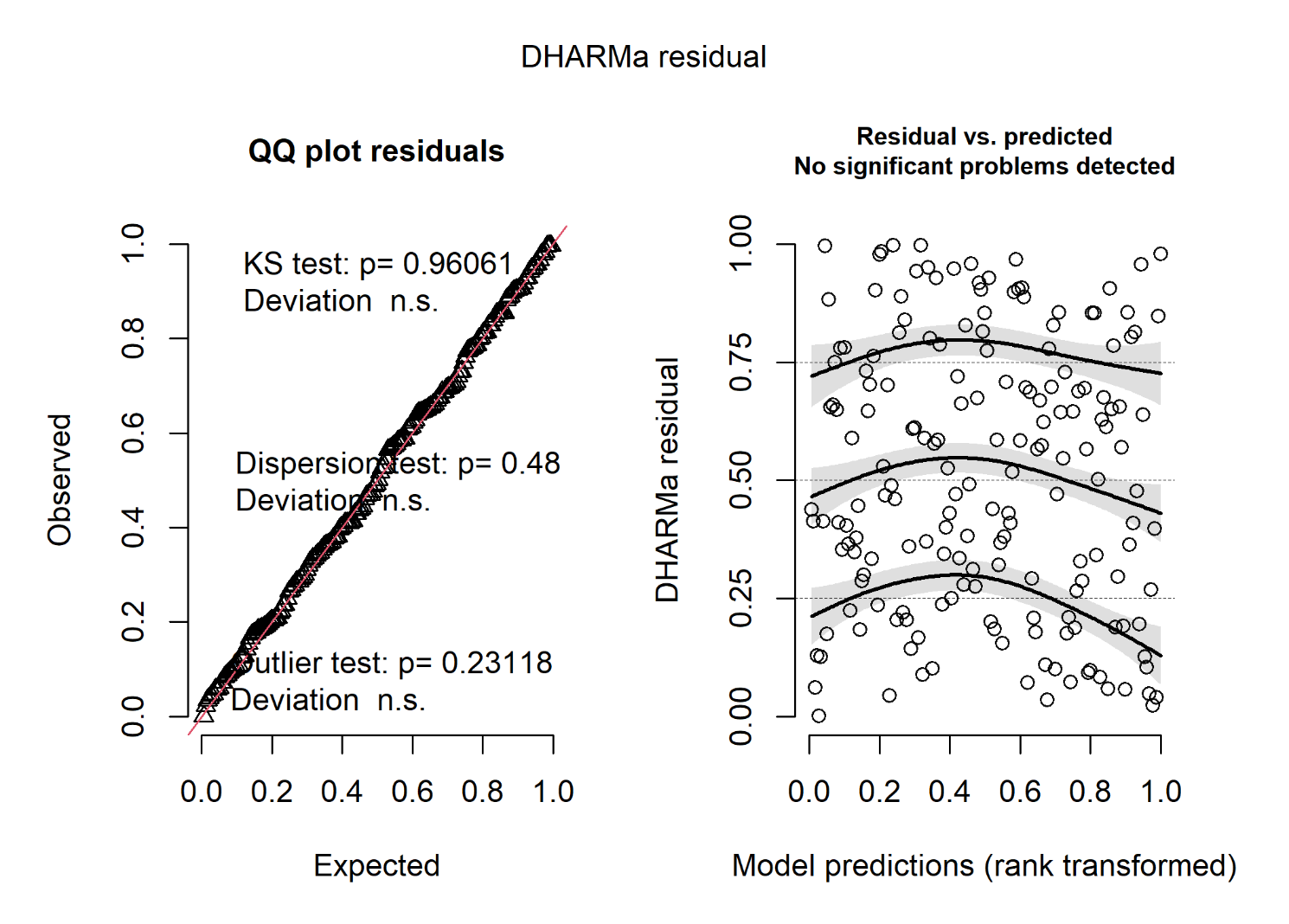


Supplementary Figure 2 Verification of model assumptions for binomial GLM (logit link)

On the occurrence of ESBL/AmpC *E. coli* in wild boar. Number of simulations = 250.


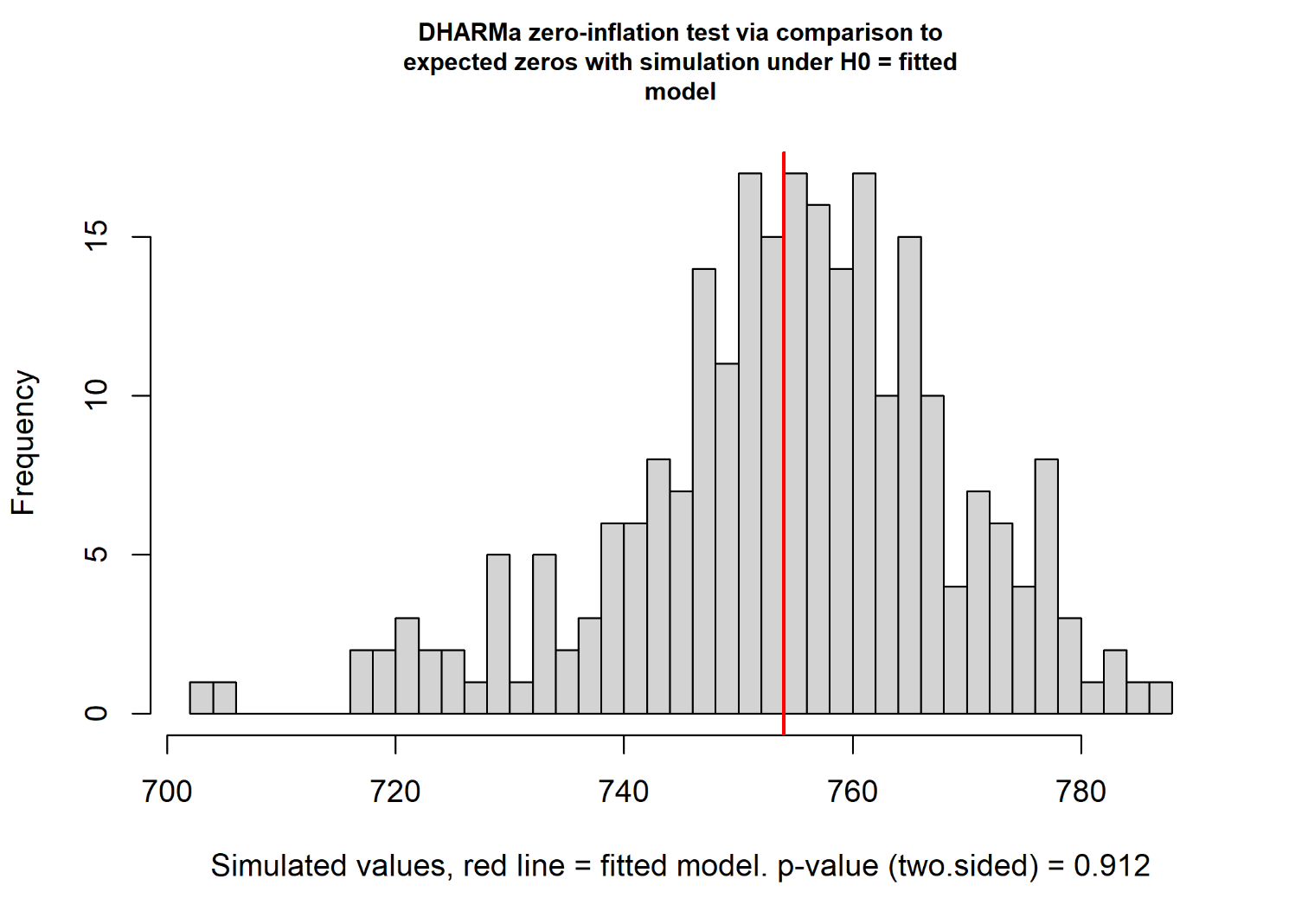


Supplementary Figure 3 Test for zero inflation shows that zero inflation is not present.


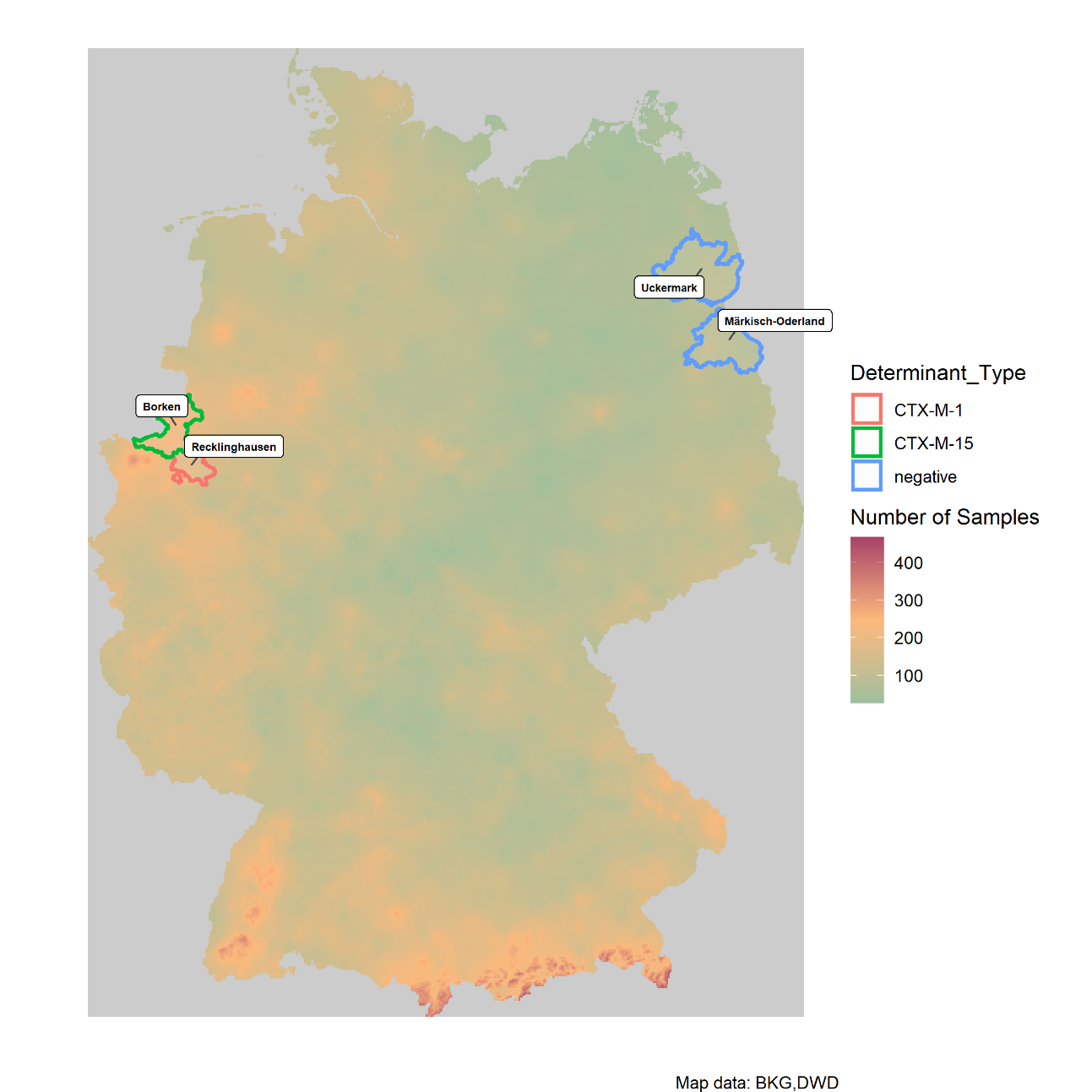


Supplementary Figure 4 Average precipitation from June 2016 and the locations where the isolates in June were sampled. The darker the color the less precipitation fell within the month. The polygons in the map show the sampled counties and the color represent the determinant type identified.


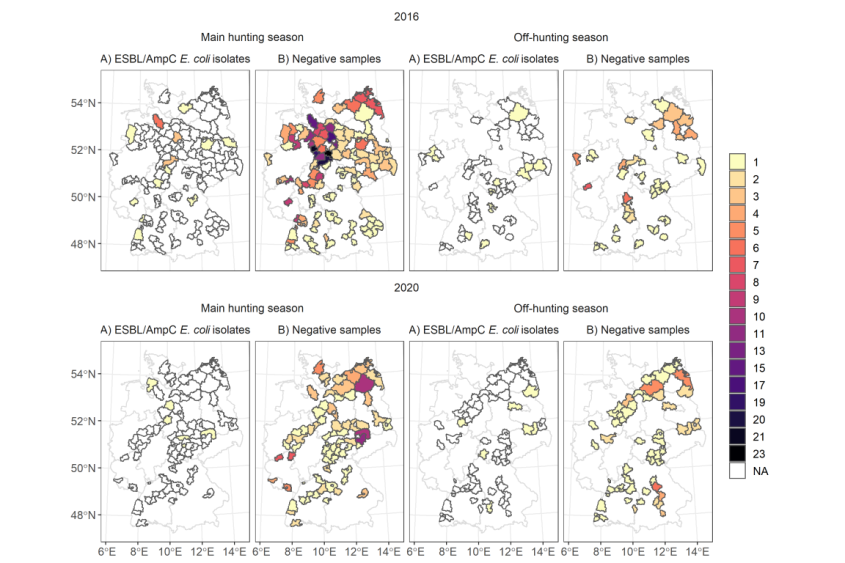


Supplementary Figure 5 Distribution of isolates and negative samples per year and hunting season.


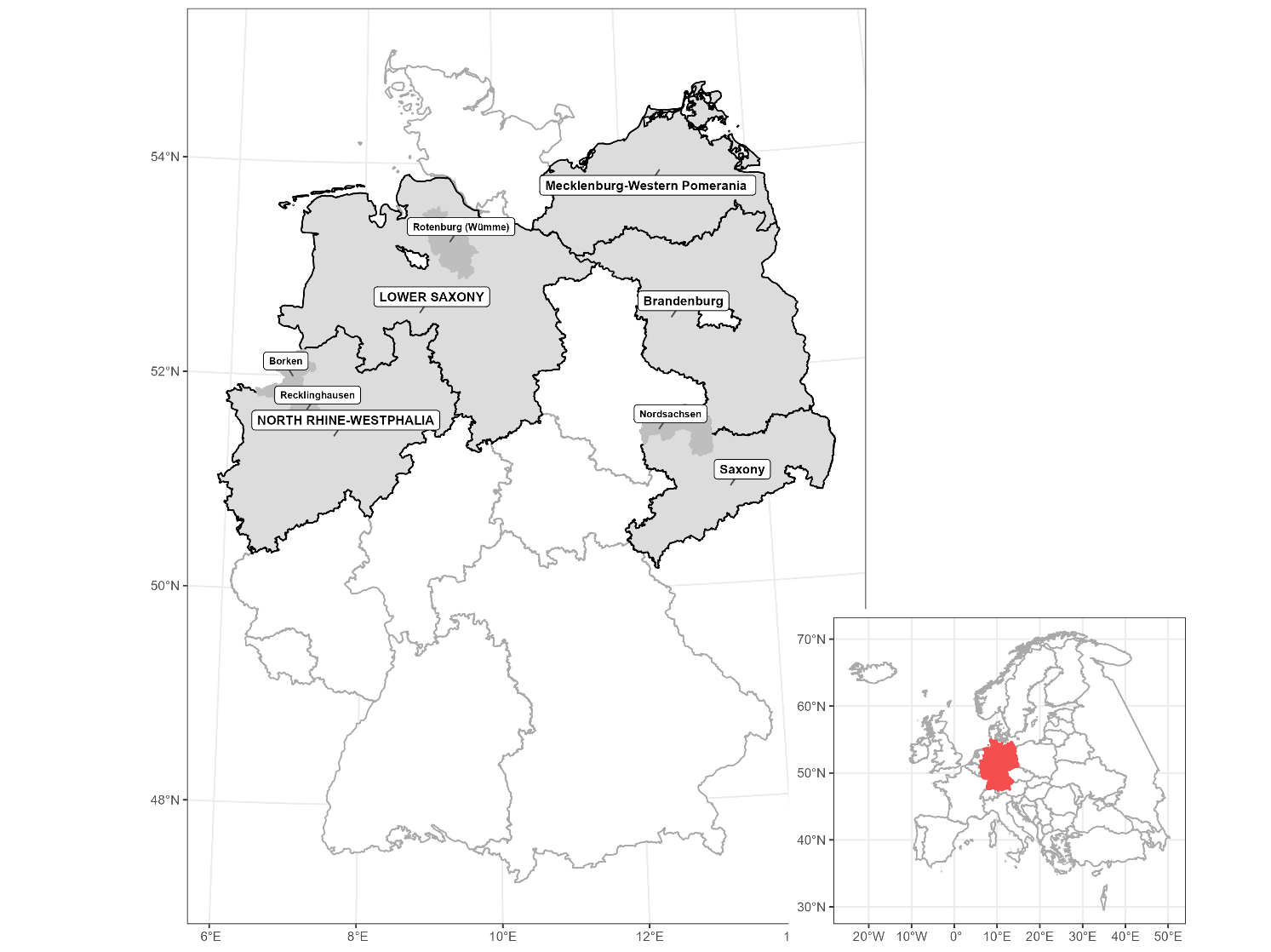


Supplementary Figure 6 Geographical location of the federal states and counties mentioned in the text.
